# A Novel Approach to Estimate the Impact of PCV20 Immunization in Children by Incorporating Indirect Effects to Generate the Number Needed to Vaccinate

**DOI:** 10.3390/vaccines13080805

**Published:** 2025-07-29

**Authors:** Mark H. Rozenbaum, Maria J. Tort, Blair Capitano, Ruth Chapman, Desmond Dillon-Murphy, Benjamin M. Althouse, Alejandro Cane

**Affiliations:** 1 Pfizer Inc., Collegeville, PA 19426, USA; maria.tort@pfizer.com (M.J.T.); blair.capitano@pfizer.com (B.C.); benjamin.althouse@pfizer.com (B.M.A.); alejandro.cane@thermofisher.com (A.C.); 2 PPD™ Evidera™ Health Economics & Market Access, Thermo Fisher Scientific, London W6 8BJ, UK; ruth.chapman@thermofisher.com (R.C.); des.dillon-murphy@thermofisher.com (D.D.-M.); 3 Information School, University of Washington, Seattle, WA 98195, USA; 4 Department of Biology, New Mexico State University, Las Cruces, NM 88003, USA

**Keywords:** pneumococcal, full value of vaccine assessment, number needed to vaccinate, PCVs, vaccination

## Abstract

**Background/Objectives:** The number needed to vaccinate (NNV) is a metric commonly used to evaluate the public health impact of a vaccine as it represents the number of individuals that must be vaccinated to prevent one case of disease. Traditional calculations may underestimate vaccine benefits by neglecting indirect effects and duration of protection (DOP), resulting in NNV overestimation. This study evaluated the NNV for the pediatric 20-valent pneumococcal conjugate (PCV20) US immunization program, as compared to PCV13, with a unique approach to NNV. **Methods:** A multi-cohort, population-based Markov model accounting for indirect effects was employed to calculate the NNV of PCV20 to avert a case of pneumococcal disease, invasive pneumococcal disease (IPD), hospitalized non-bacteremic pneumonia (NBP), ambulatory NBP, and otitis media (OM), as well as to prevent antibiotic-resistant cases and antibiotic prescriptions. **Results:** The mean NNV over a 25-year time horizon to prevent one case of pneumococcal disease was 6, with NNVs of 854 for IPD, 106 for hospitalized NBP, 25 for outpatient NBP, and 9 for OM, 11 for a course of antibiotic, and 4 for resistant disease. The mean NNV per year decreased over time, reflecting the DOP and increasing indirect effects over time. **Conclusions:** This study presents a novel approach to NNVs and shows that relatively few vaccinations are required to prevent disease. The decrease in NNV over time highlights the necessity of including DOP and indirect effects in NNV calculations, ensuring a more realistic assessment of a vaccine’s impact.

## 1. Introduction

Pneumococcal infection, caused by *Streptococcus pneumoniae*, continues to be a significant public health concern, leading to diseases, such as pneumonia, meningitis, and otitis media (OM) [1,2]. A dramatic reduction in the incidence of pneumococcal diseases has been observed over the past 20 years since the introduction of pneumococcal conjugate vaccines (PCVs) in children [3,4].

Introduced in the year 2000, the first pneumococcal conjugate vaccine (PCV7) targeted seven pneumococcal serotypes, leading to a reduction in pneumococcal disease incidence among children under five years of age, as well as among older adults due to indirect effects [3,5,6,7]. A decade later, the introduction of PCV13, which includes an additional six serotypes (1, 3, 5, 6A, 7F, and 19A) [5,6,8], brought about a further decline in invasive pneumococcal disease (IPD) rates in young children and significantly lowered the number of pneumococcal pneumonia cases and associated hospitalizations in the adult population [9,10]. Later generation, higher valency PCVs are currently available. Although the effectiveness data of higher-valency PCVs are still under assessment, these PCVs are expected to further reduce pneumococcal disease incidence [11].

To achieve widespread immunization, vaccines are implemented into national immunization programs (NIPs). In many countries, the incorporation of vaccines into an NIP is based on several criteria. For example, in the US, the Centers for Disease Control and Prevention (CDC) uses the evidence to recommendation (EtR) framework, which considers seven criteria [12]. The number needed to vaccinate (NNV), representing the number of individuals that must be vaccinated to prevent one case of disease or another outcome, can serve as a measure for the evaluation of two of these criteria: ‘benefits and harm’ and ‘resource use’. The NNV has been historically used by the Advisory Committee on Immunization Practices (ACIP) in evaluations of and recommendations for vaccines, such as Hepatitis B [13] and pneumococcal [14,15] vaccines. Furthermore, the NNV has been frequently used in the literature to quantify the effectiveness and comparative strategies of vaccination programs [16,17,18] and has been applied to various health outcomes, vaccine types, and populations [19,20].

Originating from the number needed to treat (NNT), which measures the number of patients needed to be treated to prevent one additional adverse outcome compared to a control in therapeutic contexts, the NNV is specifically designed for evaluating the impact of vaccination programs in preventative health [17]. Despite their shared origins, the NNV introduces unique complexities and differs in its calculation compared to the NNT. While the NNT is calculated as the reciprocal of the absolute risk reduction, the NNV is traditionally calculated as the inverse of the product of the annual disease incidence rate among unvaccinated individuals and the vaccine’s effectiveness [16,17,19], thereby combining vaccine effectiveness with age-specific disease incidence [19].

Traditional NNV calculations typically rely on annual risk estimates based on short-term clinical data and, thus, have a limited time horizon. This approach is appropriate for vaccines conferring protection for ≤1 year. However, many vaccines offer protection for an extended duration, including PCVs. For example, in young children, PCV13 has been shown to provide significant protection for up to 4 years following the booster dose [21]. For vaccines with a longer DOP, the traditional approach to calculating the NNV may underestimate the true value. McLaughlin et al. [18] demonstrated that, in adults, accounting for the cumulative protection of PCV13 over five years significantly reduces the NNV for preventing community-acquired pneumonia (CAP). This underscores how limiting the assumed DOP in NNV calculations can lead to an underestimation of a vaccine’s overall impact.

Furthermore, traditional NNV calculations do not account for potential indirect effects (i.e., herd effects), leading to biased estimates up to three orders of magnitude [20]. Including indirect effects in NNV calculations is crucial because vaccines may prevent cases in unvaccinated populations, which should be considered in evaluating the NNV. A significant benefit of pediatric pneumococcal vaccination is the protection it provides to older adults, contributing substantially to the overall health impact of these vaccination strategies [22,23,24,25,26]. Although the ACIP recognizes the NNV as an appropriate and intuitive summary measure it acknowledges its limitations as not all aspects are captured, such as indirect effects [27].

Incorporating both direct and indirect effects along with the DOP into NNV calculations allows for a more comprehensive understanding of the true impact of vaccination programs to support informed public health decisions and more efficient allocation of resources [16,17,18,19,20]. Therefore, this study aimed to evaluate the NNV for PCV20 in the US population across the lifespan while incorporating indirect and cumulative effects of the pediatric immunization program. In addition, scenario analyses were conducted to better understand the impact of adjusting the model time horizon and including and excluding indirect effects on the NNV calculation.

## 2. Materials and Methods

Traditional NNV calculations incorporate the annual incidence rate of a vaccine-preventable disease among unvaccinated individuals and a vaccine’s effectiveness using the following formula:

$$Traditional\;NNV=\frac{1}{Incidence\;rate\times Vaccine\;effectiveness}.$$

In this study, we used a model-based approach, enabling the incorporation of both the direct and indirect effects of vaccination over an extended time horizon to compare the NNV of the vaccine of interest (PCV20) to the standard of care (PCV13) when PCV20 was introduced rather than no vaccination. The updated NNV calculation used in this analysis was based on the approach outlined by Brission et al. [19] where

$$Updated\;NNV=\frac{Number\;vaccinated}{Cases\;prevented\;over\;time\;horizon}.$$

### 2.1. Model Structure

The model used in this study to estimate the NNV has been adapted to several countries to estimate the cost-effectiveness of PCV20 [28,29,30,31,32,33,34,35,36,37,38,39,40,41,42,43,44,45,46,47]. Briefly, this population-based, multi-cohort, Markov model simulates the US population based on predicted births over a time horizon of up to 25 years and accounts for the gradual build-up of indirect effects and the sustained protection provided by the vaccine. Direct effects were modeled based on reductions in the incidence of IPD, pneumonia, and OM in vaccinated individuals. Although pneumococcal carriage and transmission are not directly modeled, indirect effects are captured by incorporating age-specific and disease-specific reductions in disease incidence, benefiting even those who were not vaccinated. The model operated on an annual cycle, introducing new cohorts of children each year vaccinated with either PCV13 or PCV20 in a 3 + 1 dosing schedule, with four doses administered at 2, 4, 6, and 12–15 months. DOP in the model was conservatively assumed to be constant for four years after the final vaccine dose [21], and thereafter would exponentially decay reducing 10% relative to the preceding year; the maximum DOP was assumed to be 15 years.

Individuals’ transitions across the health states within each cycle were determined by vaccine strategy-specific and age-specific transition probabilities. Detailed information of the model structure and inputs is presented in Rozenbaum et al. [33].

### 2.2. Primary Analysis

The model outcomes included the NNV of PCV20 compared to PCV13 to prevent IPD and OM cases, hospitalization, and hospitalized and outpatient CAP. Additionally, the model assessed the NNV to prevent mortality and antibiotic-resistant cases prevented and antibiotic prescriptions avoided. The outcomes were analyzed over a 25-year time horizon.

Uncertainty around the base-case NNV estimates was investigated through deterministic and probabilistic sensitivity analyses. A one-way deterministic sensitivity analysis (DSA) was conducted to identify the drivers of NNV and examine uncertainty by adjusting the value of each input to the high and low range based on published data or ±20% of the base-case values in the absence of published data. A second-order stochastic probabilistic sensitivity analysis (PSA) was conducted with 1000 iterations to assess joint uncertainty in the parameter estimates using typical probability distributions following Briggs et al. [48], to generate confidence intervals around the point estimates of NNV. Confidence intervals (CIs) and standard errors (SEs) were used to inform parameter uncertainty, while upper and lower bounds (±20% of the point estimate) were applied in the absence of CIs or SEs. The values used in the DSA and PSA can be found in Rozenbaum et al. [33].

### 2.3. Scenario Analyses

Scenario analyses were conducted to show the impact of omitting certain critical factors. Firstly, the time horizon of the analysis varied between 1 and 25 years. The one-year time horizon reflects the basic NNV calculation, which only considers annual cases. Additionally, the impact of indirect effects was assessed, thereby allowing a comparison of the NNV with direct effects only versus the NNV capturing the population-level benefit.

## 3. Results

### 3.1. Primary Analysis Results

**Figure 1:**
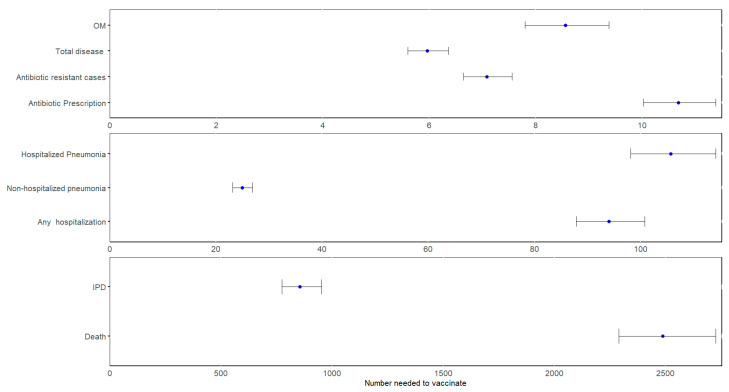
NNV for outcomes in the base case analysis. Abbreviations: IPD = invasive pneumococcal disease; OM = otitis media.

As shown in Table 1 and Figure 1, when comparing PCV20 to PCV13 over a 25-year time horizon, the mean NNV varied by outcome as follows: 85 vaccinations were needed to prevent one case of IPD, 106 for hospitalized pneumonia, 25 for non-hospitalized pneumonia, 94 for any hospitalization, 9 for OM, and 6 for any disease. The analysis also showed that 11 vaccinations were needed to prevent a course of antibiotics, and 7 to prevent a case of antibiotic-resistant disease.

### 3.2. Scenario Analysis

**Figure 2:**
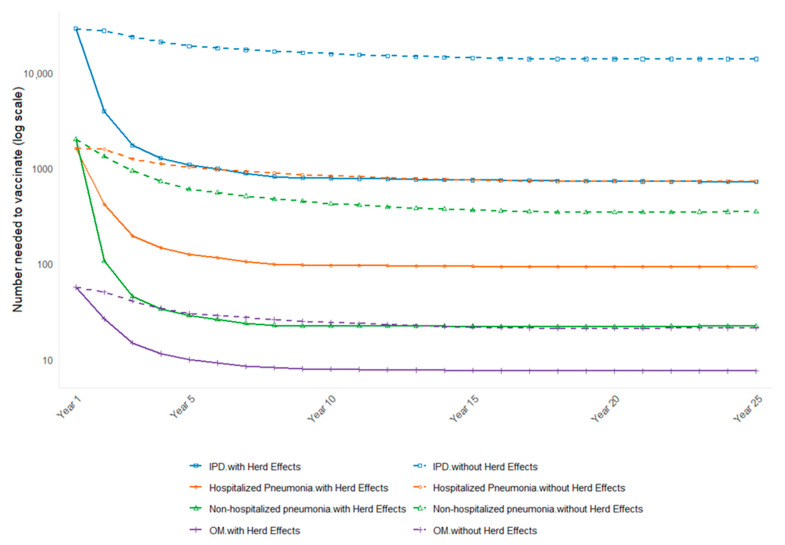
Annual number-needed-to-vaccinate (NNV) for PCV20 versus PCV13 under scenarios that include versus exclude indirect (herd) effects, by disease outcome, over a 25-year horizon. The x-axis shows calendar years following PCV20 introduction; the y-axis shows the NNV required to avert one additional case. For each outcome—(A) invasive pneumococcal disease (IPD), (B) hospitalized non-bacteremic pneumonia, (C) non-hospitalized pneumonia, and (D) otitis media (OM)—solid lines trace the base-case scenario that incorporates indirect protection, while dashed lines represent a counterfactual in which herd effects are set to zero. Abbreviations: IPD = invasive pneumococcal disease; OM = otitis media; NNV = number needed to vaccinate.

The NNVs for all outcomes decreased over time, with the highest NNV observed in the first year of the time horizon, followed by a steep decline and a plateau (Figure 2). These results reflect the pattern of the accumulation of indirect effects in the population. In every outcome, the NNV is highest in year 1, reflecting benefits limited to the directly vaccinated cohort; values decline steeply during years 2–10 as indirect protection accumulates in older, previously unvaccinated cohorts, and then plateau. Omitting indirect effects keeps the NNV elevated throughout the horizon, with the gap widening over time (e.g., total-disease NNV of 6 with indirect effects versus 23 without at year 25).

In the scenarios excluding indirect effects, the NNV is consistently higher than that in the primary analysis, although the difference is small with shorter time horizons (<10 years). For total disease, the NNV increased from 6 to 23 when indirect effects were excluded in the scenario with the 25-year time horizon while there was no difference in the scenario with a 1-year time horizon (NNV = 53) reflecting that indirect effects would gradually increase after the first year of the introduction of PCV20 in the national immunization program. The results of the scenarios assessing different time horizons and excluding indirect effects are shown in Table 2.

### 3.3. Sensitivity Analysis

**Figure 3:**
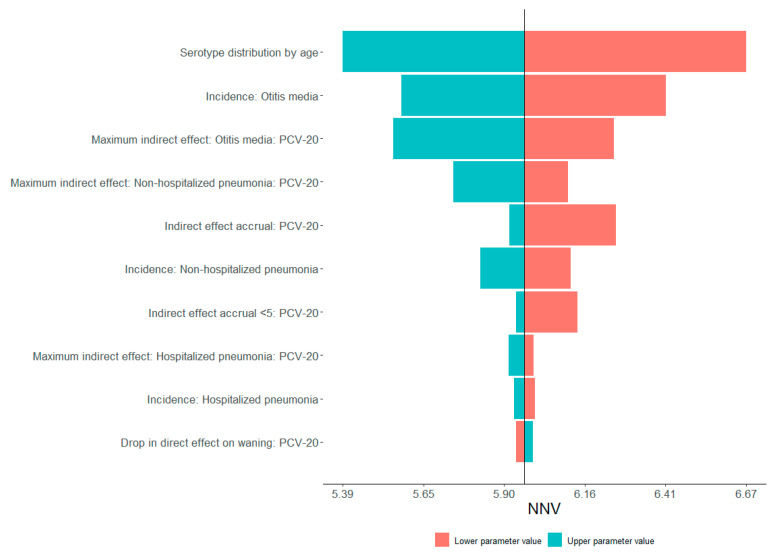
One-way deterministic sensitivity analysis (DSA) for the total-disease number-needed-to-vaccinate (NNV) when replacing PCV13 with PCV20 over a 25-year time horizon. The vertical gray line marks the base-case NNV (≈5.9). For each input, the model was rerun twice—once with the parameter set to its lower bound (salmon bars) and once with the upper bound (teal bars)—while all other inputs remained at their base-case values. Horizontal bar length therefore represents the absolute change in NNV attributable to that single parameter; longer bars denote greater influence. Parameters are ordered from top to bottom by decreasing impact. The x-axis shows the resulting NNV, ranging from 5.39 (most favorable) to 6.67 (least favorable). Abbreviations: NNV = number needed to vaccinate; PCV20 = 20-valent pneumococcal conjugate vaccine; SoC = standard of care.

The DSA revealed that the model conclusions are highly robust (Figure 3): across all plausible parameter values, the NNV varied only between 5.4 and 6.7 doses. The age-specific serotype distribution of PCV20-exclusive serotypes was the dominant driver, and higher prevalence of these serotypes reduced the NNV by ~9%, whereas lower prevalence increased it by ~13%. Baseline incidence of complex OM and the maximum indirect effect for OM and pneumonia were the next most influential factors; higher disease burden or stronger herd protection each lowered the NNV by 5–8%. Conversely, the slower accrual of indirect effects or faster waning of direct protection had modest upward pressure, but each shifted the NNV by less than one-tenth of a dose. No single epidemiological or vaccine-effect assumption was capable of overturning the overall finding that upgrading from PCV13 to PCV20 requires vaccinating about six individuals to avert one additional case of pneumococcal disease.

## 4. Discussion

Accurately calculating the NNV is crucial for understanding the true impact of vaccination programs. Traditional NNV calculations often overlook the DOP and indirect effects, leading to an underestimation of a vaccine’s long-term benefits. Incorporating these factors provides a more accurate and comprehensive assessment of vaccine effectiveness, which is essential for informed public health decisions and efficient allocation of resources. This study presents a novel approach to calculating the NNV by incorporating both direct protection and indirect effects, as well as the cumulative benefits over an extended time horizon. In this analysis, PCVs were used as a representative example to illustrate the application of this methodological approach.

Given that PCV7 and PCV13 have already significantly reduced the incidence of pneumococcal disease in the US [3,4], this analysis compared PCV20 to PCV13 rather than no vaccination to provide a more relevant assessment of the additional benefits provided by PCV20. Over a 25-year period, the NNVs to prevent one case of IPD, hospitalized and non-hospitalized pneumonia, any hospitalization, OM, any pneumococcal disease, and death were 854, 106, 25, 94, 9, 6, and 2488, respectively. Furthermore, the NNV to prevent one case of antibiotic resistance was 7, and the NNV to prevent one antibiotic prescription was 11. Sensitivity analysis results demonstrate that the NNV estimates are robust with relatively little variability in input scenarios. The outcomes included represent the key public health impact of pneumococcal vaccination and allow for the consideration of optimal vaccination strategy, and the inclusion of antibiotic-resistant cases and prescriptions goes beyond the standard outcomes for pneumococcal vaccines demonstrating the larger benefit to society.

The NNV for preventing various health outcomes with PCV20 substantially decreased with an extended time horizon and when including indirect effects. The decrease in the NNV over time highlights the necessity of including the duration of vaccine effectiveness and indirect effects in NNV calculations, ensuring a more realistic assessment of a vaccine’s impact. The importance of including an extended time horizon in NNV calculations was previously also shown by McLaughlin et al. [18], who compared two methodologies for calculating the NNV with PCV13 to prevent one case of community-acquired pneumonia (CAP) among US adults aged 65 years. However, their study is not directly comparable to our analysis. While McLaughlin et al. [18] focused on a cohort of 100,000 older adults receiving PCV13, we evaluated the broader impact of PCV20 across the entire US population using a population based model. Two other studies also examined the NNV for PCV20 in adults ≥18 years and older adults aged ≥60 or ≥65 years. However, these studies applied shorter time horizons—5 years [49] and 15 years [50]—compared to the 25-year horizon used in our study. Additionally, while our study compared the impact of PCV20 versus PCV13, previous studies used no vaccination as comparator [50]. Finally, our methodology for calculating the NNV also differed: Lewnard et al. [49] employed a per capita approach based in case reductions, whereas we used a model capturing cumulative vaccine impact over time.

Like all studies, ours has several limitations that warrant discussion. While the model captures indirect effects, it does not directly model pneumococcal carriage and transmission dynamics. Consequently, the true extent of indirect effects is unknown, which could potentially lead to an underestimation or overestimation of the population-level impacts. This limitation could affect the accuracy of our NNV estimates, particularly in reflecting the broader community benefits of vaccination. Conservatively, we assumed that a large proportion of the adult population (i.e., elderly persons previously vaccinated with PCV13, younger adults with underlying conditions previously vaccinated with PCV13 or PPV23) would not benefit from the indirect effects of childhood PCV20 vaccination at any time during the modeling horizon. In the absence of evidence on the DOP conferred by PCV20, data for PCV13 were employed [21]. Savulesu et al. [21] showed that up to 4 years after the booster dose the vaccine effectiveness of PCV13 was stable. In our model we assumed that after four years, the effectiveness would wane by 10% per year until year 15. After 15 years the effectiveness was conservatively assumed to be 0%. Currently there are no data on PCV20 DOP, which could potentially impact the conclusion on the influence of DOP time horizon. Additionally, the model approach does not consider serotype replacement as this was not observed in the US, serotype replacement could alter the profile of NNV over time [51,52]. While the model relies on US-specific epidemiological data, which may limit generalizability to other settings, the relative consistency of pneumococcal disease patterns and vaccine effects across developed countries suggests that our findings would remain directionally applicable, however, more studies are needed to estimate NNV in other countries. Although we did not explicitly model all possible disease sequelae, our inclusion of major outcomes, like IPD, pneumonia, and OM, captures the most significant public health impacts. The exclusion of rarer complications would likely make our NNV estimates conservative.

## 5. Conclusions

The results of our study strongly support the notion that the standard definition of NNV should be reconsidered for vaccines providing protection for more than one year and those conferring indirect effects. Traditional NNV calculations, which often use annual risk estimates, may underestimate the true impact of long-term vaccination programs. By incorporating cumulative effects and extending the time horizon, this study provides a more accurate and meaningful measure of vaccine effectiveness, highlighting the significant public health benefits of PCV20. Our use of a decision-analytic Markov model allows for a detailed simulation of population dynamics across multiple health outcomes and enhances the reliability and real-world applicability of the results, while the inclusion of extensive sensitivity analyses (both deterministic and probabilistic) demonstrates the robustness of the findings. Accurately estimating the NNV by incorporating cumulative, long-term public health benefits is crucial for the decision-making processes of governmental vaccine technical committees and policymakers worldwide. Our findings underscore the necessity of considering extended time horizons and indirect effects to capture the full benefits of vaccination, thus providing a more robust foundation for public health decisions.

## Figures and Tables

**Table 1:** NNV for outcomes in the base case.

Outcome	Mean NNV (95% CI ^a^)
IPD	854.32 (773.62–952.85)
Hospitalized pneumonia	105.72 (98.18–114.19)
Non-hospitalized pneumonia	24.92 (23.18–26.87)
Any hospitalization ^b^	94.08 (87.89–100.82)
OM	8.55 (7.80–9.37)
Total disease ^c^	5.96 (5.60–6.36)
Antibiotic-resistant cases	7.08 (6.64–7.55)
Antibiotic prescription	10.67 (10.02–11.38)
Death	2488.07 (2290.50–2726.50)

Abbreviations: CI = confidence interval; IPD = invasive pneumococcal disease; LY = life year; NNV = number needed to vaccinate; OM = otitis media; QALY = quality-adjusted life year; ^a^ 95% CI estimates were derived from the probabilistic sensitivity analysis; ^b^ includes all IPD cases and hospitalization pneumonia; ^c^ includes all disease outcomes listed above (IPD, hospitalized and non-hospitalized pneumonia and OM).

**Table 2:** Mean NNV for all outcomes of PCV20 versus PCV1 for scenario analysis with and without heard effects and a time horizon of 1, 10, and 25 years.

	1-Year Time Horizon			10-Year Time Horizon			25-Year Time Horizon		
	With Indirect Effects	W/o Indirect Effects	<18 with Indirect Effects	With Indirect Effects	W/o Indirect Effects	<18 with Indirect Effects	With Indirect Effects	W/o Indirect Effects	<18 with Indirect Effects
IPD	28,788	28,788	28,788	1212	19,180	11,016	854	15,726	9040
Any hospitalization ^a^	1534	1534	1534	124	980	455	94	791	370
Hospitalized pneumonia	1620	1620	1620	138	1033	475	106	833	386
Non-hospitalized pneumonia	1996	1996	1996	31	614	70	25	428	56
OM	57	57	57	11	31	11	9	25	9
Total disease ^b^	53	53	53	7	29	9	6	23	7
Resistant cases	63	63	63	9	34	11	7	26	9
Antibiotic prescriptions	95	95	95	13	51	16	11	40	13
Death	104,207	104,207	104,207	3666	92,703	40,974	2488	72,054	32,853

Abbreviations: IPD = invasive pneumococcal disease; LY = life year; NA = not available; OM = otitis media; QALY = quality-adjusted life year; ^a^ includes all IPD cases and hospitalization pneumonia; ^b^ includes all disease outcomes listed above (IPD, hospitalized and non-hospitalized pneumonia and OM).

## Data Availability

No new data were created or analyzed in this study. Data sharing is not applicable to this article.
